# Study protocol for the strategic steering, implementation, evaluation, and dissemination of the Transfer Cluster of Academic Teaching Nursing Homes in long-term care in Germany (TCALL) - a quasi-experimental longitudinal study

**DOI:** 10.1186/s12912-026-04325-4

**Published:** 2026-01-23

**Authors:** Jennifer Frense, Benedikt Preuß, Matthias Zündel, Stefan Wollnik, Heinz Rothgang

**Affiliations:** 1 SOCIUM Research Center on Inequality and Social Policy, University of Bremen, Mary‑Somerville‑Straße 5, 28359 Bremen, Germany; 2 High-Profile Area Health Sciences, University of Bremen, Bibliothekstraße 1, 28359 Bremen, Germany; 3 City University of Applied Sciences Bremen, Am Brill 2–4, 28195 Bremen, Germany; 4 Leibniz Science Campus Digital Public Health, 28359 Bremen, Germany

**Keywords:** Evaluation, Long-term care, Nursing homes, Teaching nursing homes, Quality of life, Job satisfaction, Organizational development, Mixed methods design, Quasi-experimental, Dissemination

## Abstract

**Background:**

Long-term care in Germany lags behind other countries, when it comes to adopting scientific knowledge into daily practice and vice versa. Structures for transferring technological innovations from science into practice or organizational development in nursing homes are missing, although comparable structures in the medical sector exist. Ensuring high-quality care remains one of the key challenges for the future in Germany, which can only be met by creating and implementing such transfer structures in the form of the “Transfer Cluster of Academic Teaching Nursing Homes” in order to make working in long-term care more attractive.

**Methods:**

The aim of the Transfer Cluster is (a) implementing three Academic Teaching Nursing Homes in Bremen with (b) a nationwide rollout after nine years. An explanatory sequential mixed-methods design with an embedded quasi-experimental long-term study is used for the evaluation of the “Transfer Cluster of Academic Teaching Nursing Homes” initiative. In three nursing homes, different elements of the Transfer Cluster (e.g., digitalization) will be implemented and evaluated (intervention group). The main outcomes of the summative evaluation are job satisfaction of employees and quality of care of residents. The formative evaluation includes research diaries and focus groups. Results will be compared with three matched nursing homes as a control group. Dissemination material will be developed and a concept for the nationwide rollout will be derived.

**Discussion:**

Long-term care faces different challenges in the next years, including the realization and connectivity to research, especially to digitalization and the growing decline in staff. The close exchange with employees of the Academic Teaching Nursing Homes and their management provides important insights into everyday practice and leads to development of innovations which are really needed and therefore more accepted by employees and are tailored to their working conditions. Clear strength of the “Transfer Cluster of Academic Teaching Nursing Homes” initiative is the duration of up to nine years and a nationwide rollout which is thought of from the beginning. A successful nationwide rollout of Academic Teaching Nursing Homes will be a great milestone in nursing science and practice.

**Trial registration:**

German Clinical Trials Register DRKS00035292 (Date of registration: December 9, 2024).

**Supplementary Information:**

The online version contains supplementary material available at 10.1186/s12912-026-04325-4.

## Introduction

Long-term care (LTC) in Germany lags behind other countries, e.g., the Netherlands or the USA, when it comes to structures that combine science, teaching and care itself. This leads to the fact that transferring innovations from science into practice or organizational development in nursing homes takes too much time [1–3]. This may be related to the fact that professionalization and academization of care came up late in Germany when compared to the Netherlands or the USA, and scientific culture and structures in LTC are in the process of construction [4]. Further ongoing challenges for nursing homes such as technological and other related health innovations and aggregated care needs are also present in everyday care [1].

To face these ongoing challenges and to enable the implementation of technological, social and organizational innovations in everyday care situations, it is essential to create transfer structures in LTC. In the medical sector, university hospitals and academic teaching hospitals offer the opportunity to carry out teaching and research directly in everyday health care. This provides a direct transfer path from research via teaching to healthcare – and vice versa, impulses from healthcare practice flow back into research and teaching. Internationally, similar concepts such as the *Living Lab in Ageing and Long-Term Care* in the Netherlands [5, 6] or the *Teaching Nursing Homes* in the USA and Norway [7, 8] have been established in LTC for more than 25 years.

These concepts are described as a possible perspective for improving the transfer and quality of care in Germany [9]. Additionally, there are initial concepts for nursing homes in the project *Nursing Practice and Science in the Care of People with Dementia in Long-Term Care through the Dementia Living Lab Approach* (PraWiDem), which, however, relates to people with dementia only and also has a limited duration of just three years [10]. A comprehensive concept for LTC is still missing, although such a “transfer structure” would also be important in Germany and would constitute a crucial component for ensuring high-quality LTC.

The innovative core idea of the “Transfer Cluster of Academic Teaching Nursing Homes”^1^ initiative is to create a Cluster of Academic Teaching Nursing Homes in order to reconcile research, teaching, and daily care-giving, and thus enabling a two-way transfer. The TCALL initiative, funded up to nine years by the Federal Ministry of Research, Technology and Space, aims to build a foundation for a new, continuously science-based care culture in LTC. In the first phase of TCALL, three nursing homes will be restructured into Academic Teaching Nursing Homes (ATNH). Subsequently, this structure will also be rolled out, first to the federal state of Bremen and finally to Germany. TCALL contributes to strengthening LTC proactively in the field of education and training, care quality, and job satisfaction by providing a space for sustainable innovation structure and culture. This newly created space allows to identify, test, and subsequently implement innovations into everyday LTC, which have been evaluated positively before. As TCALL also aims to disseminate the established structures widely in Germany before the initiative ends, TCALL has a significant scientific and societal impact, particularly when considering the recent challenges in LTC. The TCALL initiative will develop approaches to ensure best LTC in the future and to address general societal challenges such as demographic change as well as upcoming challenges like robotics and artificial intelligence (AI) [11].

## Background

### Situation in Germany

Ensuring high-quality care remains one of the central challenges of the future in Germany, which can only be met by creating transfer structures between science and practice in order to make LTC more attractive. This includes improved working conditions, introducing technological innovations across LTC, and adapting internal workflows in LTC through human resources development and organizational development, as well as continuing education and training content and structures. However, there are currently no effective and sustainable structures to support nursing homes in implementing the necessary organizational and technological innovations. On the one hand, nursing homes in Germany have not yet established a consistent scientific culture, including appropriate continuing education structures. This is because training as a nurse is nonacademic in Germany and nursing homes do not have a cooperation with universities so far. On the other hand, knowledge about their acceptance, effectiveness, and efficiency is still limited, although technological innovations have been promoted for many years [12, 13]. Another fact is that science usually has not a direct access to nursing homes and the real working conditions. This might lead to asking the wrong questions for researchers and providers who focus on the development of technological innovations that do not solve the real-world problems in LTC. Therefore, implementing successful innovations in standard care rarely succeeds and the process is lengthy [2, 3]. Various studies show that evaluations under laboratory conditions and the establishment of showrooms with technical solutions for practical transfer alone are not sufficient, as there is a lack of high-quality evaluations [12, 13] and the development of innovations often bypasses practical needs [14]. In addition, the participatory involvement of care-givers is often lacking, which is an important condition for integrating innovations into daily routines [15]. Also the legal framework does neither provide refinancing for the introduction of digital care innovations nor for follow-up costs [16]. Human resources and organizational development processes also require long-term testing and continuous development in nursing homes under real conditions. Besides these facts, the demographic change is ongoing while the number of employees in care continues to decrease [17] which carries a risk for decreasing quality in caring.

### Aims and structure of the TCALL initiative

The TCALL initiative is funded up to nine years by the German Federal Ministry of Research, Technology and Space and is a cooperation between the University of Bremen, the City University of Applied Science Bremen, the Integrated Health Campus Bremen and three nursing homes as practical partners (one from Johanniter and two from Caritas^2^). TCALL combines various sub-projects to achieve the vision of a Transfer Cluster of Academic Teaching Nursing Homes. This vision aims to create a direct transfer pathway from research via training to care – and vice versa, from care practice and the identification of problems back to research and training which address these problems. This is achieved by introducing innovations (social and technological) into LTC across the board and adapting internal work processes in LTC accordingly through personnel and organizational development, as well as further developed training content and structures. TCALL offers the opportunity to implement innovations directly in everyday care in the ATNH, which is not possible in conventional research projects and under laboratory conditions. These real-life conditions will also lead to feedback loop effects for science so that new and relevant research questions can be derived and tackled. In order to support these processes, special staff was hired and introduced in the three ATNH. These transformation and innovation agents (TIAs)^3^ are a special feature in the TCALL initiative, and they are responsible for a regular exchange between nursing homes and research partners. The TIAs form the interface to the ATNH and ensure a trustful cooperation. They will support all measures within TCALL to ensure that processes run smoothly and to reduce the workload of employees in the participating nursing homes.

**Fig. 1:**
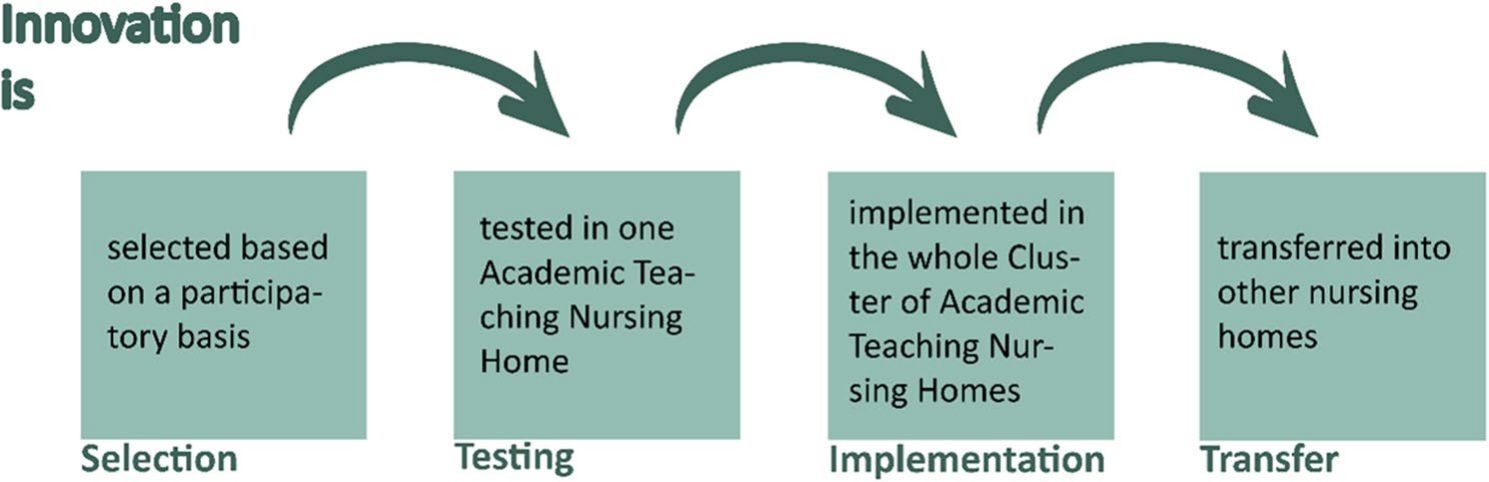
Transfer path of TCALL

In summary, TCALL aims at a double innovation structure: Firstly, the diverse technical, social and organizational innovations will be participatory selected, tested in one ATNH, evaluated, and implemented in the whole Cluster of Academic Teaching Nursing Homes. Subsequently these innovations will be prepared for transfer into other nursing homes. This applied transfer pathway (Fig. 1) is based on a framework for evaluating technical innovations in LTC [18–20]. Secondly, a transfer structure will be created enabling recursive feedback between the nursing homes, the innovators and later the regulatory authorities, the mutual and joint development and implementation of new technical products and organizational structures. In this way, the TCALL initiative contributes to improving the quality of care and working conditions in the LTC sector and to achieving financial sustainability for the ATNH, by bringing together all stakeholders involved in the provision of LTC.

After a phase of planning and preparation in 2023 (start-up phase), the first sub-projects started in December 2023 and January 2024. The first one (Development of a decentralized learning infrastructure) aims to design and develop a learning infrastructure for LTC close to the workplace including a combination of different learning concepts. The second (Center for Coordination and Transfer), which is the subject of this study protocol, is responsible for the overall evaluation, strategic steering and dissemination of the entire TCALL initiative as well as for the development of strategies for a nationwide rollout.

**Fig. 2:**
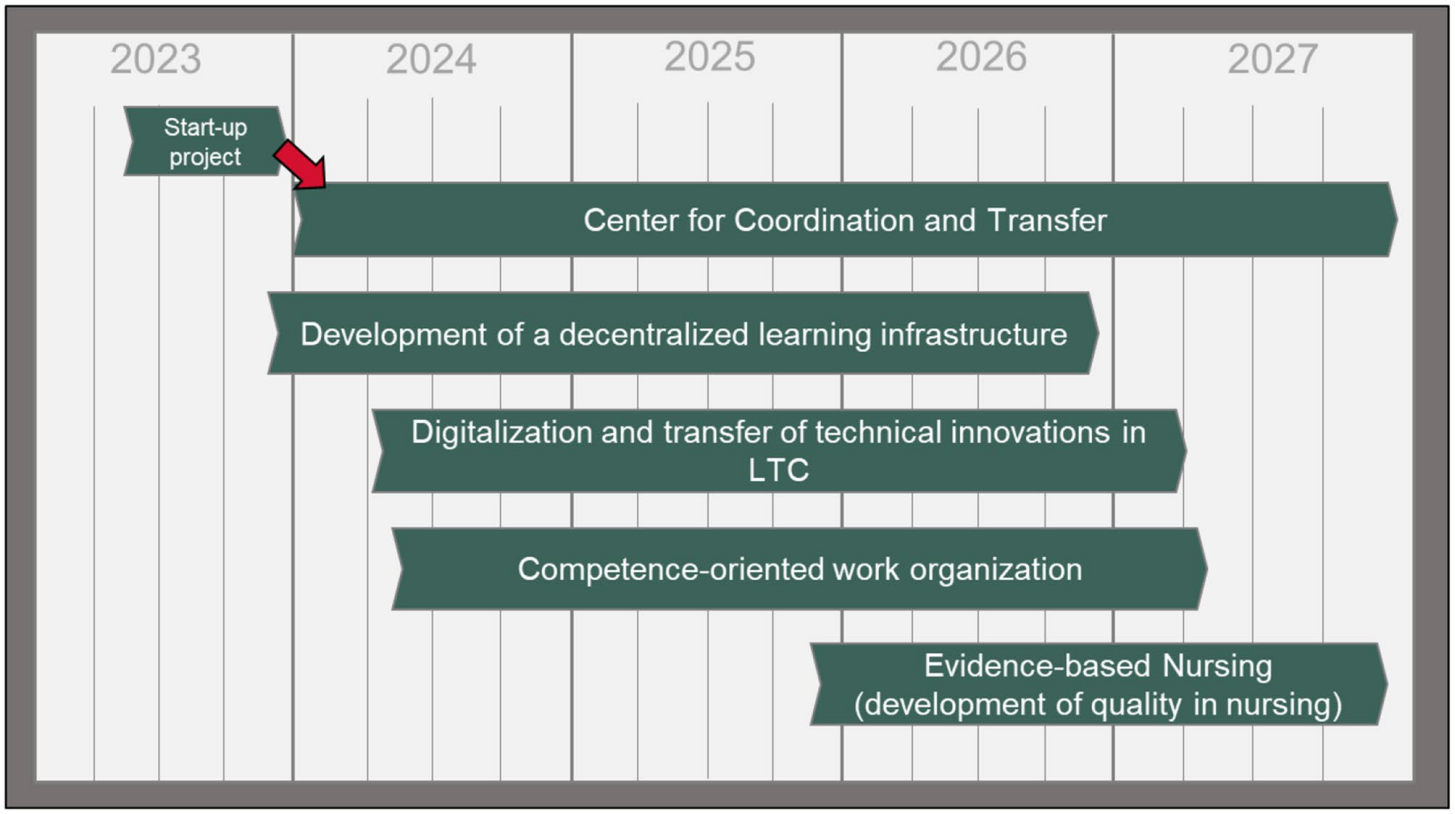
Timetable of sub-projects of the TCALL initiative

Two more sub-projects started in 2024: “Digitalization and transfer of technical innovations in LTC” aims to select and test digital technologies, while “Competence-oriented work-organization” aims to implement new organizational and personnel structures in nursing homes based on the qualification levels of the employees. The objective of “Evidence-based Nursing” is the establishment of developed instruments and measures in the context of evidence-based nursing in participatory cooperation with the ATNH and is planned for the end of 2025. The timetable for all sub-projects in TCALL until the end of 2027 can be seen in Fig. 2.

### Aims of “Center for Coordination and Transfer”

**Fig. 3:**
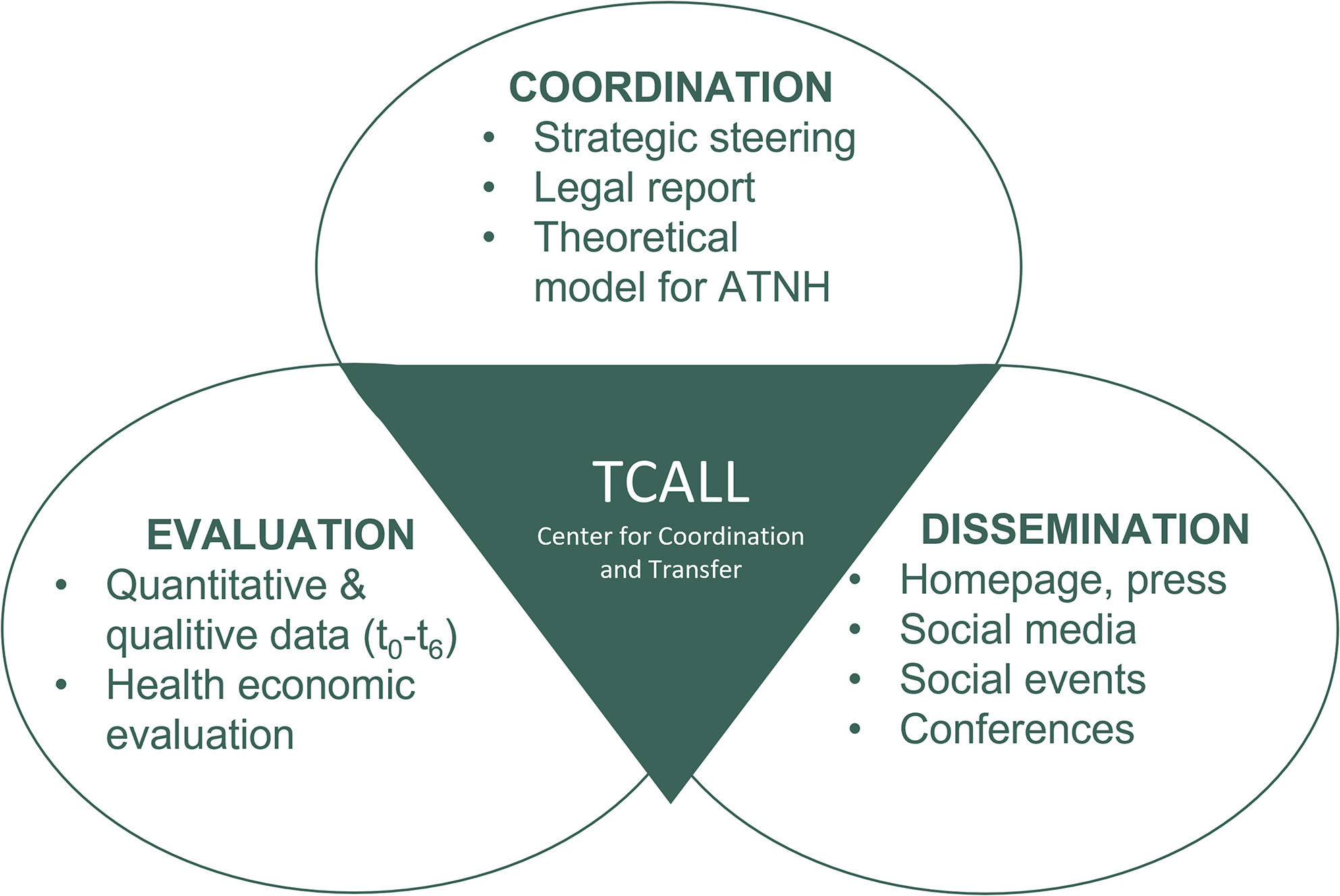
Products of the sub-project Center for Coordination and Transfer

The “Center for Coordination and Transfer”, which is the subject of this study protocol, is responsible for the coordination, evaluation and dissemination of the overall TCALL initiative. It is made up of three main aspects (Fig. 3):

1. Strategic steering, including a legal report and development of a theoretical model for ATNH,
2. Evaluation of the TCALL initiative and,
3. Dissemination of innovations that have been tested in practice and, in the event of a positive assessment, implemented on a broad scale and thus achieve both care-related and scientific utilization.

The overall goal of these three aspects is to permanently consolidate the structures developed by the TCALL initiative on a national level by bringing together actors with similar interests on a political, scientific, economic and social level beyond the federal state of Bremen and its region. The assessment of the impact and measurability of the influences on care recipients and employees through an overall evaluation of the TCALL initiative is necessary, firstly, to draw scientific conclusions from the initiative, and, secondly, to legitimize the continuation of the initiative.

## Methods and analysis

### Strategic steering - Coordination

The “Center for Coordination and Transfer” is concerned with coordinating the various sub-projects of the TCALL initiative and aligning their content. Regular meetings are held for this purpose. In addition, it acts as a data management center and works closely with the Competence Center for Clinical Trials Bremen, which acts as a trust center for the data. As a result, all TCALL sub-projects can use the data in pseudonymized form. To provide strategic guidance, a model for the structure of ATNH will be developed and a legal report will be commissioned by a legal expert on what part of the legal framework needs to be adapted to allow for a nationwide rollout, which will serve as the basis for a nationwide rollout.

#### Data collection

The study protocol follows the SPIRIT reporting guidelines for study protocols for clinical trials. Outcomes will be measured with reliable, valid and pretested instruments (Table 1). Most used questionnaires are free to use and do not need any license except for “The Neuropsychiatric Inventory Nursing Home Version (NPI-NH)” and the *“*Adult Social Care Outcomes Toolkit (ASCOT-SCT4)”. Licenses in both cases were obtained and permission for use is confirmed. Ethical approval was confirmed by the ethics committee of the German Society of Nursing Science in October 4th, 2024. Baseline data collection in the intervention group started in November 11th, 2024 and is ongoing, follow-up data collection continues at six-month intervals, and the end of the first study phase is planned for 2027. Questionnaires for employees and residents are completed digitally, with the exception of the Adult Social Care Outcomes Toolkit (ASCOT-SCT4) [21], which is paper-based. Data collection of residents’ files, structural data of institutions and data from quality audits will be retrieved by the staff of the University of Bremen and the City University of Applied Sciences in Bremen. All data collection will take place in the intervention nursing homes and matched control groups. Data analysis is pseudonymized.

#### Data management

A comprehensive data protection concept has been developed and approved in advance by the legal office of the University of Bremen. It clarifies data processing, the rights of participants, and technical and organizational measures in order to ensure the secure and confidential collection, processing, and storage of data. Data is stored at the Competence Center for Clinical Trials Bremen which acts as a trust center. Access to pseudonymized data is limited to research staff from the University of Bremen and the City University of Applied Sciences in Bremen.

### Evaluation

#### Study design and participants

**Fig. 4:**
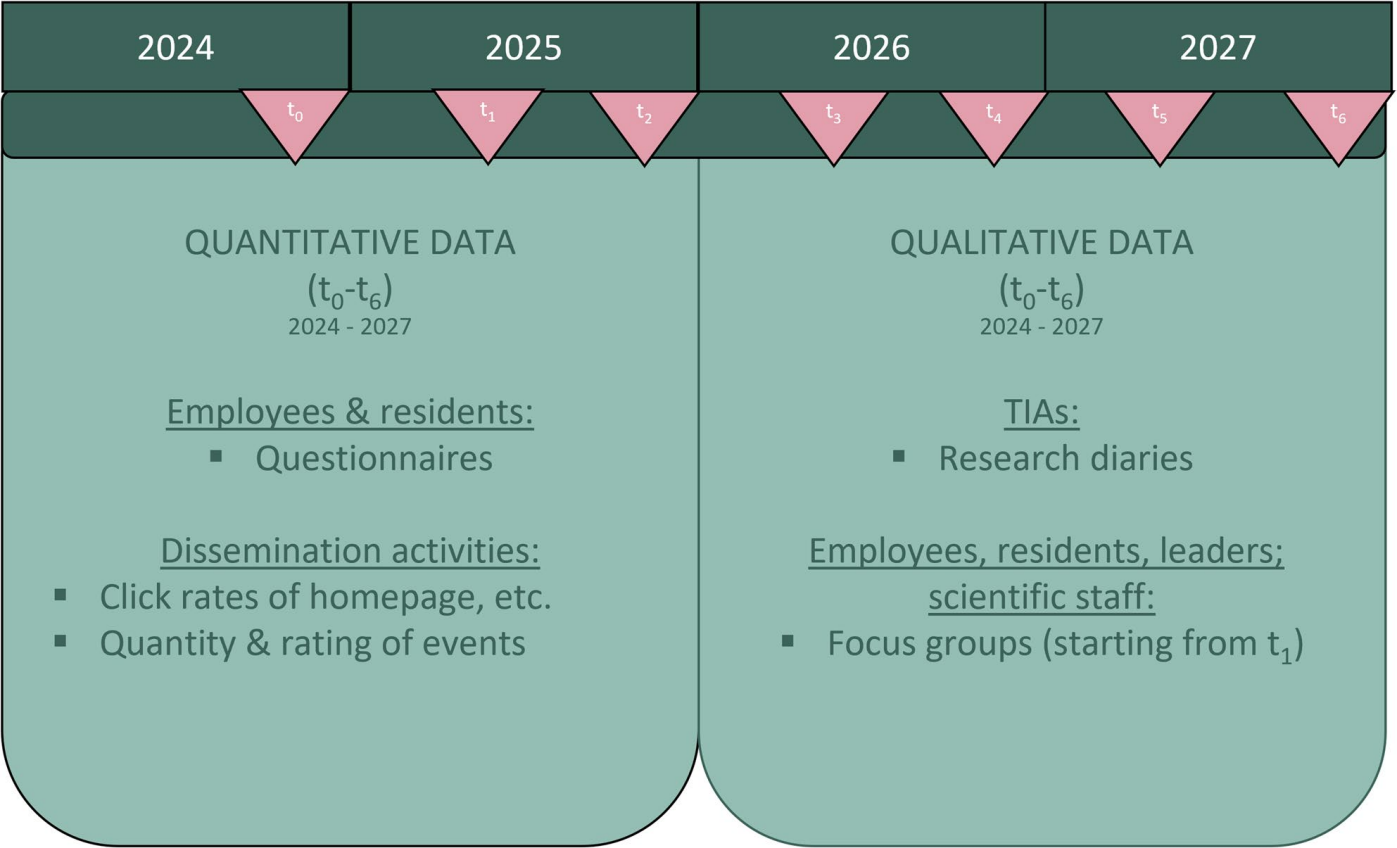
Quantitative and qualitative data

The basic methodology is a systemic evaluation approach [22], which combines both qualitative and quantitative research elements. The intended restructuring in the nursing homes can affect different, sometimes ethically relevant impact dimensions such as the quality of care for highly vulnerable people but also working conditions and processes. These dimensions will be captured using quantitative (standardized questionnaires) and qualitative methods such as focus groups and research diaries to gain further insights from residents, employees, nursing home management and TIAs concerning the implementation process of the several elements within the TCALL initiative (Fig. 4). For a successful implementation it is essential to fully understand rejecting or driving behaviors of all participating members in order to adapt the implementation process. This can lead to minor or major adaptions in single or multiple parts of the TCALL initiative. Additionally, qualitative methods support the participative, co-creative approach and will ensure that all participants (but especially the employees) feel valued and help changing and shaping the restructuring processes in nursing homes. In contrast to laboratory conditions, it is of great importance to address essential disruptive factors in everyday care immediately in order not to jeopardize the initiative and to keep employees and management motivated. An explanatory sequential mixed-methods design with an embedded quasi-experimental long-term study is used for the evaluation of this complex intervention to ensure that aspects of the quantitative results are interpreted correctly and to have reference points when contradictory results occur [23]. The study design also takes into account that confounding factors can influence the assessment of the outcomes. An important factor here has been the pandemic situation of the last few years, which has had a major impact on the care situation in LTC [24, 25] as well as changes in care policy, such as the implementation of new staffing ratios and their financing (Paragraph 113c of the German Social Code, Part 11). In order to avoid bias in the evaluation of the initiative, which may be associated with future changes, a matched control group is integrated into the evaluation.

The evaluation aims to measure the acceptance, effectiveness and efficiency of the TCALL initiative, thus providing internal quality assurance as well as external evidence of the added value of the TCALL initiative. In addition, initial approaches for dissemination and a sustainable nationwide rollout will be recorded.

The evaluation contains summative and formative components. The summative evaluation focuses on outcomes at two different levels: (i) employees and (ii) residents, with the most important outcome for employees being job satisfaction and for residents being quality of care. The formative evaluation aims to investigate the process of implementation of new structures, the acceptance of these structures and the achievement of goals for individual aspects in the TCALL initiative. Additionally, a health-economic evaluation with a cost-benefit analysis is planned.

##### Eligibility criteria

Participants in this study can be either employees or residents in selected nursing homes (three ATNH and three matched controls). Inclusion criteria for employees is to have worked in the nursing home for at least four weeks prior to the start of the survey. This is intended to prevent positive and negative effects on the employees’ job satisfaction that may arise when starting a new job. No further exclusion criteria is used. Residents can participate in the study if the residents themselves or an authorized person (such as a legal guardian) confirm written informed consent. For ethical reasons care recipients at the end of their lives are excluded, and those who have been living in the nursing home for less than six weeks. This is due to the fact that a change of setting and living situation might have a positive or negative impact on the quality of life, depending on the previous situation at home.

##### Recruitment

Three nursing homes, which already report a high-quality standard and have a high and qualified staff ratio in order to avoid overloading of the nursing homes, have been selected to become the first ATNH (convenience sample). They were chosen as good and trustful relations with these nursing homes and their management already existed. The nursing homes are located in Bremen and are non-profit organizations. The nursing homes of the control group will be matched to the intervention group (number of beds, costs for residents and located in Bremen). The recruitment for the control group is ongoing.

Participation in the study is voluntary and based on written informed consent. Residents of the intervention group will be recruited by management, employees and TIAs. Management and employees of the intervention group have been informed in person at information events held by the study team in the participating nursing homes. The aim of these events was to inform management and staff of the nursing homes about the TCALL initiative and to enlist support from them. The control group will be recruited and informed by the scientific staff of the University of Bremen and the management of the control nursing homes.

##### Sample size

A sample size will not be calculated. The cooperating nursing homes are a convenience sample and well-chosen due to the good and trustful relationship. The aim for recruitment is to involve as many employees and residents as possible. Challenges for recruitment of employees and their compliance is the already high workload which will be already stressed by new tasks and changes in work organization in terms of becoming an ATNH. Residents are considered as a vulnerable group and should be as little burdened as possible. Another challenge here is the fact that many residents are not able to speak and confirm consent for themselves due to dementia or other diseases. In these cases, an authorized person (e.g., a legal guardian) has to confirm consent, which is not under the supervision of university staff.

#### Summative evaluation

As briefly described above, the evaluation focuses on different outcomes at two different levels: (i) employees and (ii) residents, but on non-intended effects as well. Changes due to the complex intervention are expected in job satisfaction of employees in the ATNH compared to control nursing homes. Additionally, there are several changes expected for employees concerning organizational commitment, mental workload or sickness rates and the use of temporary employment agencies. For residents it is expected that the complex intervention will alter quality of care in the ATNH compared to control nursing homes. Further outcomes include the number of nursing home-sensitive hospital diagnoses and apathy symptoms. A full list of all outcomes and instruments is shown in Table 1. All outcomes are measured at baseline, at multiple follow-ups at six-month intervals and at the end of the study (in this case the end of the first phase in 2027). Outcomes are measured via (standardized) questionnaires and/or external assessment by a nurse, and an analysis of resident files and routine data of nursing homes. Sociodemographic data of employees and residents will be collected and analyzed. A secondary data analysis of obligatory quality indicators and external quality audits will be conducted. These internal and external quality indicators (see supplementary material additional file 1) are mandatory in Germany since 2022 and are reported publicly semi-annually. Internal quality indicators include 15 self-assessed items regarding three themes collected by nursing homes and their employees. External quality audits are structured in six themes with 24 items and are collected from assessors and evaluators on behalf of the LTC insurance fund (e.g., Medizinische Dienste/medicproof GmbH).

**Table 1:** Overview of outcome and instruments for employees and residents

Outcome	Instruments (Source)
**Employees**	
Job satisfaction	EXQ [26]
Organizational commitment of employees	
Individual commitment of employees	
Collective commitment of employees	
Mental workload	COPSOQ [27]
Employees’ influence and development opportunities	
Employees’ skills	Self-developed
Readiness of digital technology	Self-developed according to [28]
Use of digital technology	Self-developed based on [29]
Sickness presenteeism and absenteeism	Self-developed
*Indicators of a high workload*:	
i. Sickness rate	Routine data
ii. Staff turnover	
iii. Use of temporary employment agencies	
**Residents**	
Quality of care	Obligatory and publicly reported quality indicators and external quality audits
Number of nursing home-sensitive hospital diagnosis	List of nursing home-sensitive hospital conditions [30]
Apathy symptoms	AES [31]
Quality of life	ASCOT-SCT4 [21], QUALIDEM [32–34]
Behavioral and psychological symptoms of dementia	NPI-NH [35]
*Complications and adverse events in persons in need of care*:	
i. Hospital admissions	Routine data
ii. Emergency medical care	
iii. Falls with injuries	
iv. Pressure ulcer acquired in the facility	
v. Use of psychotropic drugs	
vi. Deprivation of liberty measures	

##### Outcomes employees

*Job satisfaction* is measured using the first item of the Employee Experience Questionnaire (EXQ) [26]. This tool allows to obtain a holistic picture of employee experience at work, which describes major perceptions, attitudes and behaviors of employees in organizations. The score ranges from 1 (completely unsatisfied) to 7 (completely satisfied). Higher scores indicate better job satisfaction.

###### Organizational commitment of employees, individual commitment of employees, collective commitment of employees

The EXQ is also used for the following outcomes: organizational commitment of employees, individual commitment of employees, collective commitment of employees. Again, scores range from 1 (no agreement at all) to 7 (full agreement), and higher scores indicate better commitment.

###### Mental workload, employees’ influence and development opportunities

The German version of the Copenhagen Psychological Questionnaire (COPSOQ) is utilized for the measurement of mental workload, the employees’ influence and development opportunities [27]. Four main topics are addressed, personal and workplace details, details of work and activities, questions about work and health, and at last free-text options. The scale ranges from 5 (always) to 0 (never/nearly never). Higher scores indicate a higher mental workload and higher influence and development opportunities.

###### Employees’ skills, readiness for and use of digital technology and presenteeism and absenteeism

A self-developed survey to measure employees’ skills is used. Included questions address qualification, advanced training or other acquired skills and years of professional experience. Readiness for digital technologies includes a short scale according to Neyer et al. [28]. Use of technology is measured via a self-developed questionnaire based on Braeseke et al. [29]. Sickness presenteeism and absenteeism is measured via three self-developed questions.

###### Indicators of a high workload

Due to the complex organizational change in staff, changes in indicators for a high workload, like sickness rate, staff turnover and the use of temporary employment agencies are expected at end of the study. To some extent, it is possible that during the restructuring process of staff deployment and shortly after implementation, the indicators for a high workload show an increase instead of a decrease. These non-intended effects are based on the fact that learning and getting used to new workflows initially leads to additional work and only later to positive effects (J-curve effect).

##### Outcome residents

###### Quality of life

Quality of life will be assessed either using the Adult Social Care Outcomes Toolkit (ASCOT-SCT4) [21] or QUALIDEM (Quality of life in dementia) for people with mild or severe dementia, if residents’ abilities or cognitive skills are not sufficient to complete the ASCOT-SCT4 (due to dementia or other diseases). If QUALIDEM is used, a familiar care-giver will first evaluate the resident’s grade of dementia via the Global Deterioration Scale (GDS) [36] and afterwards complete the QUALIDEM questionnaire for a resident. An overall score and scores for each subscale will be calculated. Depending on an overall scale or a subscale the score assumes higher or lower quality of life for people in need of long-term care [32].

###### Nursing home-sensitive hospital admissions

Nursing home-sensitive diagnoses are defined as diagnoses, which could have been preventable in at least 70% of all hospitalizations with such a diagnosis, if care in nursing homes had been adequate before. Bohnet-Joschko et al. (2022) published a list of 58 several diagnoses which are assigned to nursing home-sensitive hospital admissions [30]. We will collect some basic information about residents, including hospital admissions, to compare these with the list of Bohnet-Joschko et al. (2022).

###### Apathy symptoms

The German version of the Apathy Evaluation Scale (AES) is used to assess apathy symptoms. Apathy can occur as an independent syndrome or is usually associated with other clinical disorders. The AES is a syndrome-independent scale which claims to assess levels of apathy in several disorders. The score ranges from 0 (not applicable at all) to 4 (very applicable). Higher scores assume few to no symptoms [31, 37].

###### Behavioral and psychological symptoms of dementia

The Neuropsychiatric Inventory Nursing Home Version (NPI-NH) is used to characterize the neuropsychiatric symptoms and psychology of patients with Alzheimer’s disease or other forms of dementia for people living in nursing homes. The NPI-NH includes ten behavioral areas and two types of neurovegetative variations. Scores are made for frequency (range 1 - 4 (rarely to very often)), severity (1 - 3 (mild to severe)), occupational disruptiveness and care-giver stress (0 - 5 (not at all to very severely or extremely)). Calculating is based on the author’s recommendations [35].

###### Quality of care in nursing homes

The quality of care is evaluated via the results of internal quality indicators and external quality audits (see supplementary material additional file 1). The reports of quality indicators and quality audits are mandatory and publicly available.

*Complications and adverse events for people in need of care* include hospital admissions, emergency medical care, falls with injuries, pressure ulcer acquired in the facility, use of psychotropic drugs, and deprivation of liberty measures. Routine data of nursing homes are used to assess changes in complications and adverse events. Routine data are collected by nursing homes in any case.

#### Formative evaluation

The formative evaluation aims to illustrate the development of structures, the acceptance of the new structures, and the achievement of specific aspects of the TCALL initiative, as well as to reflect on the dissemination concept. To this end, research diaries are maintained, focus groups and event reflections are conducted, and digital marketing metrics are analyzed.

##### Research diaries

Specially assigned TCALL staff in nursing homes (TIA) regularly keep research diaries and reflect on their daily routines, on daily work routine of employees and on care situation of residents. With these diaries actual conditions and needs can be determined and care-giving in nursing homes can be monitored continuously. Research diaries include key questions which are answered every two weeks and focus questions on special topics whenever needed and useful (Table 2). Results of the research diaries will be clustered and summarized into fact sheets. These fact sheets will be presented to employees and residents and finally discussed and validated in workshops (six months interval).

**Table 2:** Questions for research diaries

**Key questions (two weeks response frequency)**	
1	What were your experiences in the last two weeks in the ATNH?
2	What are your current tasks as a TIA?
3	What challenges do you have to face as a TIA?
4	Have you observed any changes in the ATNH in the last two weeks? (Please explain your answer briefly)
5	What else would you like to tell us?
**Focus questions (three months response frequency)**	
	*Self-reflection of TIAs concerning skills*
1	What further knowledge is required to perform better as a TIA?
	*Integration in ATNH*
2	What is helpful to be accepted as a TIA in your nursing home?
3	To what extent have these conditions been met so far?
	*Development of the ATNH*
4	What developments regarding the ATNH have been observed in the facilities up to date?
5	What ATNH-related topics is the organization currently working on?
6	How are the developments in relation to the ATNH accepted and how do you notice it?

##### Focus groups

Focus groups will be conducted with employees, residents, head of nursing homes, TIAs and research staff at least once a year. Perceptions of one’s own role, the acceleration and process of introduced innovations is reflected here. Results are discussed and validated by participants and will be analyzed for further evaluation on themes like process evaluation, acceptance and success or failure of implemented innovations.

#### Health economic evaluation

The health economic evaluation aims first, to calculate the expenses of the intervention and second, to relate the outcomes of the intervention to its costs (cost effectiveness analyses). In doing so, it is essential to distinguish between the costs of the interventions and the costs of the study, particularly the costs of the evaluation of the initiative. While TIAs are necessary for sustainable ATNH and the costs of ongoing innovations and their evaluation are also part of the intervention, the evaluation of the TCALL initiative as a whole and the dissemination are study costs, which have to be treated differently. For the measurement of effects data from the summative evaluation is used. Costs do not only include direct intervention costs, but also the costs of TIAs and all measures necessary for a sustainable structure of the ATNH. As far as outcomes can be measured in monetary terms (e.g., reduction in sickness rate) cost-benefit analyses are conducted from the perspective of the nursing home. With respect to the reduction of hospital admissions cost-benefit analyses from a societal perspective are added.

#### Statistical analysis

The statistical analysis examines the changes in the outcomes in both groups, i.e., employees and residents, in t0 compared to t1-t6 and checks whether these changes differ between the intervention and control groups (difference-in-difference approach). Via correlation analyses we also analyze correlations between different outcome measures as e.g., job satisfaction and quality of care. Confounders will be controlled via regression analyses.

Information conducted within the formative evaluation especially from focus groups will be analyzed descriptively. Click rates and social media views are counted and analyzed with several digital analysis tools and programs to evaluate the dissemination activities.

### Dissemination

To realize TCALL’s vision of a nationwide rollout, respective dissemination activities are necessary. TCALL’s communication and dissemination strategy includes an identification of relevant stakeholders (Table 3) for TCALL’s (external) scientific communication and the corresponding communication goals.

Persons in need of care and their relatives are primarily interested in an increase in the quality of care and the quality of life for residents, which is to be achieved through technical and organizational innovations and human resources development, resulting from the close integration of science and practice. Nursing staff benefit from TCALL as the innovations make everyday practice easier. Moreover, education and training methods and tools will be improved. For service providers and health insurance companies, TCALLs results of the health economic evaluation may be interesting. With respect to LTC and health policy, the focus is on the prospect of both more efficient and more effective structures and processes in care provision. From a scientific perspective, TCALL offers an excellent opportunity to analyze selected innovations during their application in everyday care.

**Table 3:** Overview stakeholders

Stakeholders
Persons in need of long-term care
Care-givers
People working in education of care-givers (e.g. nursing trainer)
Service providers
Financing agencies (e.g. LTC funds)
Politicians/Political institutions
Researchers (in nursing science)
Media
Other providers (doctors, therapists)

These nine stakeholders require target group-specific communication measures. In addition, certain actors have a particularly important role to play in the realization of the project vision in the form of a sustainable (nationwide) rollout of TCALL. These actors are divided into two groups accordingly, namely the group of multipliers and the group of decision-makers. The multipliers are separate actors from the individual TCALL interest groups who pass on project content to other interested parties, multipliers and decision-makers and, if necessary, also actively convince them to support the initiative. This group of stakeholders is able to support the strategy implementation process. This group includes, for example, influential organizational actors such as interest groups in the healthcare sector.

Decision-makers are actors with great potential for political action in health policy or care practice (e.g. political institutions, politicians; other actors involved in financing the project such as health insurance companies or their associations). The focus of addressing decision-makers is on increasing both the quality of care and cost efficiency.

Before multipliers and decision-makers can be specifically addressed through communication activities, they must be identified. In the course, a database of relevant persons is built up and continuously expanded. The strategic TCALL network created in this way is an important component of the communication and dissemination strategy of the TCALL initiative.

## Discussion

### Summary and placement in the literature

LTC faces different challenges in the next years, including the realization and connectivity to research, especially to digitalization and the growing decline in staff. Making the nursing profession attractive again, especially in LTC, and creating an innovative transfer structure between science and practice working in both ways is the primary objective of TCALL with the development and implementation of ATNH in the federal state of Bremen, and finally in Germany. Other countries, like the USA or Netherlands, have already set off approaches to renew LTC and promote exchange between science and practice a long time ago. The Living Lab in Ageing and Long-Term Care by Maastricht University in the Netherlands successfully created a collaboration between scientists, care providers, and educators in LTC in order to improve quality of life of older people and their families, quality of care and quality of work for nursing staff. This collaboration has been established for 25 years now, and main factors for success are Linking Pins and interdisciplinary approaches [5]. TCALL comes into these factors by introducing TIAs and interdisciplinary supervision. Similar work and goals can be found in Teaching Nursing Homes in the USA or Norway where partnerships between service and academia include benchmarks of nursing home professional learning environment, interdisciplinary education and practice as well as research and dissemination of evidence-based practices. Teaching Nursing Homes in the USA started in the 1980s in order to improve resident outcomes and to foster research among other things. Few schools of nursing homes and medicine have maintained this model over the past 15 years. However, up to date, there has been little research concerning effectiveness in terms of whether Teaching Nursing Homes can potentially enhance research training opportunities in the health professions and contribute significantly to better outcomes for residents [38]. TCALL addresses exactly this point and takes a formative and summative evaluation into account. In addition, the course is set for national expansion by asking and clarifying legal questions during the development and implementation of ATNH.

### Strengths and limitations

The vision of the TCALL initiative is to restructure the provision of care by strengthening the transfer path between science and practice. A great advantage and chance for success is the long duration of the initiative, up to nine years, which enables an adequate testing of the different innovations, time for adaption and an accompanying implementation. This is nearly never given in Germany, especially in research projects. This long period holds great potential to really improve care, the quality of care, working conditions and the quality of life. A lot of things which are often not done in research due to time or cost limits, like e.g., several process adaptions, is possible in TCALL. Furthermore, the close exchange with employees of the ATNH and their management provides important insights into everyday practice and leads to development of innovations which are really needed and therefore more accepted by employees and are directly tailored to their working conditions. In many studies the implementation of innovations fails as nursing home staff has no capacities to integrate these innovations in everyday routine. The new created decentralized learning infrastructure and the TIAs, funded by the TCALL initiative, are the additional resource needed to overcome this problem. The TIAs are a clear strength as they act as an interface between science and practice. There is often massive criticism because practice partners are not involved in the process, or developments from research are not relevant to the day-to-day work of nursing staff in long-term care. The explicit mission of the TIAs is to gain and share insights and observations from the field so that these important aspects of employees and ATNH can be taken into account. This will lead to improved processes concerning the selection of (technical) innovations and their implementation.

Another strength is that the dissemination strategies for consolidation of sustainable ATNH and for a nationwide rollout are thought of from the beginning with the idea to establish a publicly funded and legally anchored concept for ATNH in Germany after nine years, which will be a great milestone in nursing science and nursing practice. If successful, the concept for ATNH could even be transferred to and implemented in other European and Western Countries to meet the demand of high-quality care with a simultaneous improvement in working conditions to reach a high attractiveness of the nursing profession.

There are few limitations in this evaluation. As the planned interventions in the TCALL initiative entail massive changes, the intervention facilities were chosen on a self-selective basis and include only nursing homes which already offer a high quality in care and high work standards (convenience sample). This might have an impact on implementation, results and overall success or failure. Moreover, a convenience sample indicates that the sample may not be typical of the population as a whole and the findings may not apply to other groups [39]. Another limitation is that at this stage, it remains unclear, if transferability to other nursing homes is possible or whether major adjustments are required.

Data collection concerning residents is carried out by nurses which results in an additional workload for them which in turn may lead to refusal or lack of commitment to the study. It can also affect data collection, which carries the risk of a high dropout rate.

The evaluation is performed by the study team itself, in detail by the coordination team, and not externally. The advantage is that the team is very familiar with the complex intervention and its implementation and therefore the interpretation of results is easier and can be used for process management and development directly. However, the self-evaluation lacks external proof of effectiveness, particularly with regard to the generalizability of the results for wider dissemination [40].

## Trial status

Recruitment for the control group is ongoing, data collection for t0 is ongoing at date of submission.

## Supplementary Information

Below is the link to the electronic supplementary material.

Supplementary Material 1

## Data Availability

No datasets were generated or analysed during the current study.

## Footnotes

1 German translation and acronym: Transfercluster Akademischer Lehrpflegeeinrichtungen in der Langzeitpflege” (TCALL).

2 Definition: Johanniter and Caritas are the names of the religious responsible body.

3 Definition of TIA: as a nursing specialist with a Bachelor’s degree in nursing science, a TIA is responsible for the connections between nursing homes and research staff. TIAs are employed by the universities but work most of their time in the nursing homes.

## Abbrevations

TCALL: Acronym for the project “Transfercluster Akademischer Lehrpflegeeinrichtungen in der Langzeitpflege” – Transfer Cluster of Academic Teaching Nursing Homes
LTC: Long-term care
TIA: Transformation and innovation agent (Transformations- und Innovationsagent)
ATNH: Academic Teaching Nursing Home
COPSOQ: Copenhagen Psychological Questionnaire
EXQ: Employee Experience Questionnaire
NPI-NH: Neuropsychiatric Inventory Nursing Home Version
ASCOT-SCT4: Adult Social Care Outcomes Toolkit
QUALIDEM: Quality of life in dementia
AES: Apathy Evaluation Scale
GDS: Global Deterioration Scale
