# Supplementary material for: Study protocol for the strategic steering, implementation, evaluation, and dissemination of the Transfer Cluster of Academic Teaching Nursing Homes in long-term care in Germany (TCALL) - a quasi-experimental longitudinal study
Source: BMC Nurs. 2026 Jan 23;25:248. doi: 10.1186/s12912-026-04325-4 (PMC12998273; doi:10.1186/s12912-026-04325-4)
Supplement: Supplementary file 1 — Supplementary Material 1 [file 12912_2026_4325_MOESM1_ESM.docx]

Additional file 1: Obligatory and publicly reported internal quality indicators and results of external quality audits

| **Internal quality indicators (15 items in total) regarding three themes:** |
| --- |
| 1. Maintaining and promoting independence (e.g., in mobility), |
| 2. Protection against health hazards and stress (e.g., in the development of pressure ulcers, falls, unintended weight reduction), |
| 3. Support for specific needs (e.g., timeliness of the pain assessment, interaction with cognitively impaired residents) |
| **External quality audits (24 items in total) regarding the six themes:** |
| 1. Support with mobility and self‑care (e.g., support with eating and drinking, mobility, continence loss, continence promotion, personal hygiene, taking medication), |
| 2. Support in coping with the demands and stresses of illness and therapy (e.g., taking medication, pain management, wound care), |
| 3. Support in organizing everyday life and social contacts (e.g., impaired sensory perception, structuring the day, occupation and communication, nocturnal care), |
| 4. Support in special needs and care situations (e.g., support during the settling‑in phase after moving in, transition management during hospitalization, support for residents with challenging behavior, use of measures involving deprivation of liberty) |
| 5. Cross-demand professional requirements (e.g., defense against risks and hazards, biography-oriented support, compliance with hygiene requirements, provision of aids, protection of personal rights and integrity |
| 6. Internal organization and quality management (e.g., qualification and performance of tasks by the responsible care professional, supporting residents and their relatives during the terminal phase, measures to prevent and remedy quality deficits) |
